# Clinical presentation, treatment patterns, and outcomes of colorectal cancer patients at Tikur Anbessa Specialized Hospital in Addis Ababa, Ethiopia: A prospective cohort study

**DOI:** 10.1002/cnr2.1869

**Published:** 2023-07-15

**Authors:** Girum Tessema Zingeta, Yohannes T. Worku, Assefa Getachew, Jilcha Diribi Feyisa, Hawi Furgassa, Winini Belay, Tariku Mengesha, Ahmedin Jemal, Mathewos Assefa

**Affiliations:** ^1^ Department of Oncology, School of Medicine Addis Ababa University Addis Ababa Ethiopia; ^2^ Department of Radiology, School of Medicine Addis Ababa University Addis Ababa Ethiopia; ^3^ Department of Oncology Saint Paul Hospital Millennium Medical College Addis Ababa Ethiopia; ^4^ Department of Reproductive Health and Health Service Management, School of Public Health Addis Ababa University Addis Ababa Ethiopia; ^5^ Department of Epidemiology St. Peter Specialized Hospital Addis Ababa Ethiopia; ^6^ Department of Surveillance and Health Services Research American Cancer Society Atlanta Georgia USA

**Keywords:** colorectal cancer, Ethiopia, survival, treatment pattern

## Abstract

**Background:**

Colorectal cancer (CRC) is the third most common cause of cancer death in both genders worldwide.

**Aims:**

This study aimed to evaluate the outcomes and prognostic factors of CRC patients at Tikur Anbessa Specialized Hospital in Ethiopia.

**Methods and Results:**

A prospective cohort study was conducted on 209 patients from January 2020 to September 2022. Kaplan–Meier curves and bivariate and multivariate Cox regression analyses were used to analyze overall and progression‐free survival, with a significance value of *P* < .05. Results showed an overall mortality rate was 67.46% (95% confidence interval [CI]: 61.0–74.0), while the 1‐year overall survival (OS) rate was 63.16% (95% CI: 56.23–69.29), with a median follow‐up duration of 20 months. The median OS and progression‐free survival times were 17 and 11 months, respectively. Age above 40 years (hazard ratio [HR] = 1.53, 1.02–2.29, *p* < .040), lower educational level (high school and below) (HR = 2.20, 1.24–3.90, *p* < .007), poor performance status (HR = 1.60, 1.03–2.48, *p* < .035), Hgb ≤12.5 g/dL (HR = 1.55, 1.03–2.08, *p* < .035), T‐4 disease (HR = 6.05, 2.28–16.02, *p* < .000), and metastases at diagnosis (HR = 8.53, 3.77–19.25, *p* < .000) were all associated with poorer survival.

**Conclusion:**

These findings suggest that poor survival of CRC patients in Ethiopia is largely due to advanced stage of the disease and lack of timely treatment, and highlight the urgent need for improved access to cancer treatment in the region.

## 1 INTRODUCTION

Noncommunicable diseases (NCDs), also known as chronic diseases, are responsible for 74% of all deaths worldwide, killing a total of 41 million people annually. NCDs include a range of medical conditions that are long‐lasting and having some form of progression over time. The most common ones being cardiovascular disease and cancer which are on the rise globally.^1^, ^2^ Cancer now kills one out of every six people worldwide, more than HIV/AIDS, TB, and malaria combined.^3^ In 2020, there were 10 million cancer deaths and 19.3 million new cancer cases.^4^ The burden of cancer is expected to increase to 27.5 million new cases and 16.2 million cancer deaths by 2040, with 65% of cases occurring in low‐ and middle‐income countries (LMIC).^5^ In both genders combined, colorectal cancer (CRC) is the third most frequent cancer worldwide, with an estimated 1.5 million new cases and it has the second‐highest mortality rate, 576 858 deaths in 2020. In males, it is the third most common in terms of incidence and mortality; however, in females, it is the third most common in terms of incidence and second in terms of mortality.^4^ According to a major Austrian study, men are twice as likely as women to develop CRC.^6^ LMICs account for 70% of all CRC‐related deaths.^7^ In Sub‐Saharan Africa (SSA), the crude incidence rate is 4.04 per 100 000, with a male to female ratio of 1.2:1.^8^ The incidence of CRC in Ethiopia is rising, with an estimated rate of 8.5 and 6.3 per 100 000 for men and women, respectively.^4^ In Addis Ababa, CRC is the most common cancer among men, accounting for 12.4% of all cancers, and the fourth most common cancer among women, accounting for 5.4% of all cancers.^9^ CRC also accounts for 12% of cancer cases at the oncology ward at Tikur Anbessa Specialized Hospital (TASH), and 7.7% of all cancer cases in Addis Ababa.^10^, ^11^

The incidence of CRC is increasing among young people of all races, particularly African Americans under the age of 50, who often present with more advanced tumors.^12^ In the United States, CRC in the young population is typically more aggressive and advanced at diagnosis. The most notable increase in incidence between the ages of 40 and 44.^13^ A study in Ghana found that the average age of people with the first CRC diagnosis was 54 ± 16.8 years, and the most frequently reported symptoms were weight loss (44.80%), rectal bleeding (39.82%), and abdominal pain (38.91%).^14^ Additionally, a 5‐year prospective study in Nigeria revealed that the majority of CRC patients at first diagnosis were between the ages of 51 and 60, and that 31% of patients were 40 years or younger.^15^

In CRC, the primary treatment options are surgery, chemotherapy (CT), targeted agents, and radiotherapy (RT), but the choice largely depends on the site of the tumor, stage at presentation, individual patient factors, and increasingly, its molecular subtype.^16^ Over time, systemic treatment for CRC has evolved from 5‐fluorouracil (5 FU) to combination regimens involving 5‐FU, oxaliplatin, irinotecan, or both, as well as the introduction of targeted agents for those with metastatic settings.^16^, ^17^ CRC is a heterogenous and complex disease that exhibits varying behaviors depending on its unique molecular pathophysiology and various molecular biomarkers can be prognostic or predictive.^18^ Recent studies have focused on CRC molecular classification in order to develop molecularly defined subgroups for prognostic and treatment purposes. The consensus molecular subtype (CMS) is a transcriptome‐based classification of CRC that is often utilized and includes 4 subtypes: CMS1 for microsatellite instability, CMS2 for chromosomal instability, CMS3 for metabolic dysregulation, and CMS4 for mesenchymal activation. CMS4 has the worst prognosis, while CMS1 has a favorable prognosis.^19^, ^20^ Recent studies have also focused on molecular classification in order to develop molecularly defined subgroups for treatment purpose.^17^ Anti‐epidermal growth factor receptor (anti‐EGFR) monoclonal antibodies, such as cetuximab and panitumumab, are used to treat metastatic cancer that expresses EGFR. Tumors with RAS and BRAF mutations, on the other hand, are not sensitive to these anti‐EGFR agents.^21^, ^22^ Similarly, 5‐FU‐based traditional CT are ineffective against tumors with a high mutation burden and microsatellite instability; instead, immunotherapies such as pembrolizumab are more effective.^23^, ^24^ The use of RT also changed the course of CRC, particularly for rectal cancer.^25^

Colorectal cancer mortality varies between countries based on human development index and racial characteristics, which is linked to the stage of disease at presentation, patient health‐seeking behavior, and treatment accessibility. In high‐income countries, mortality is decreasing, while in LMIC, it is increasing. SSA has the highest CRC mortality to incidence ratio in the world.^7^ Studies have shown that recurrence‐free survival and the stage of the disease are the most important predictors of CRC survival. In China, the three‐ and five‐year survival rates were 74% and 68%, respectively,^26^ while in Europe, the 1, 3, and 5‐year survival rates were 90%, 70%, and 63%, respectively.^27^

Despite advances in personalized cancer treatment in developed countries, cancer management in developing nations remains limited due to the lack of access to surgical care, availability of CT and RT, limited availability of healthcare professionals, and associated high costs.^17^, ^28^ This resulted in most patients in developing countries to continue having poor clinical outcomes and survival.^29^

Only 27 of the 43 SSA nations have structured cancer registration systems; data quality varies, and national coverage is limited.^7^ Significant numbers of CRC patients do not receive standard recommended therapies in most low‐income countries, including Ethiopia. This is attributable to health‐seeking behavior and, more crucially, the scarcity of integrated care.^30^ In Ghana, a retrospective hospital‐based study showed that the median OS of CRC patients over a five‐year period was 15 months, with an OS rate of 16.0%.^29^ In Nigeria, between 2013 and 2017; 71% of patients diagnosed with CRC underwent the necessary surgical procedure and 50.5% received the recommended CT. The median OS for those with stage III and IV CRC was 24 and 10.5 months, respectively.^31^ However, in Ethiopia; the treatment outcomes, including, progression free survival (PFS), recurrence, and prognostic factors associated with OS of CRC patients were not studied. To address this gap in knowledge, we conducted a study at TASH in Addis Ababa, Ethiopia, to assess the treatment patterns and outcomes, as well as factors associated with OS of CRC patients. This is a pioneer prospective study of its kind that provides an understanding of the treatment patterns and outcomes of CRC patients, which will give insight into the gaps in the current treatment interventions and inform future strategies for quality comprehensive cancer care in the country. It will have important implications for the development of a multi‐level assignment in the prevention, diagnosis, management, and follow‐up of CRC patients and serve as a solid foundation for future investigators.

## 2 METHODS

### 2.1 Study setting and participants

This study was conducted in the adult oncology department of TASH, Addis Ababa University, which houses the only RT center in Ethiopia. It is the only hospital that provides comprehensive cancer care, including surgery, CT, RT, and palliative care services.

### 2.2 Study design

Institutional‐based unmatched prospective cohort study was conducted to assess clinical presentation, treatment pattern, and outcome of CRC patients at TASH. Participants had clinical assessments, which included confirmatory diagnostic tests, appropriate primary and metastatic staging. Treatment details were documented, and patient outcomes were monitored. Patients' PFS and OS were determined through clinical follow‐up and telephone interviews.

### 2.3 Source population

All CRC patients seen at TASH oncology center during the study period.

### 2.4 Study population

All CRC patients who started treatment at TASH oncology center from January 1, 2020 to September 10, 2022.

### 2.5 Inclusion and exclusion criteria

All eligible localized and metastatic CRC patients who visited TASH's oncology center were recruited for the study. The inclusion criteria included all biopsy‐confirmed cases of CRC who began treatment and subsequent follow‐up at TASH Oncology Center with adequate clinical examination, baseline laboratories such as whole blood cell count (CBC), liver function tests (LFTs), renal function tests (RFTs) and imaging such as abdominopelvic computerized tomography scan for colon primary and pelvic magnetic resonance imaging for rectal primary and baseline colonoscopy for non‐emergency cases. Patients with adequate metastatic work up with abdominal sonography for all cases and chest x‐ray (CXR) for pulmonary metastatic evaluation; if CXR is suspicious that should be confirmed with chest computerized tomography scan. All eligible patients starting from January 1, 2020, were prospectively included in the study until August 10, 2021. Patients with an ambiguous diagnosis, inadequate baseline CBC, LFT, RFT laboratory workup, inadequate diagnostic and metastatic staging workup, and those who did not consent to be part of the study were excluded from this study.

### 2.6 Sample size

This study included all eligible patients with a diagnosis of CRC. A total of 415 CRC patients were observed at the oncology center's outpatient department, with 209 meeting the criteria for inclusion and forming the final sample size.

### 2.7 Study variables

The study variables consisted of dependent variables, such as treatment outcome (alive, death, disease progression), and independent variables, such as sociodemographic factors (age, gender, marital status, educational status, habits, monthly income, comorbidities), clinical and pathologic characteristics, including, performance status, hemoglobin level, histology, stage at presentation, and types of treatments received (surgery, CT, and RT).

### 2.8 Operational definitions

We operationalized the stages of CRC at presentation as stages I and II (early stage) or stages III and IV (advanced stage) based on the American Joint Committee on Cancer's (AJCC) 8th Edition (2017) and assessed general well‐being and activities of daily life with the Eastern Cooperative Oncology Group (ECOG) performance scale. PFS was the time between treatment and recurrence, progression or death and OS was the proportion of patients who were alive after some period among the study population.

### 2.9 Data collection tools and procedures

Three oncology resident physicians underwent two‐day training for the purpose of data collection and extraction techniques. The principal investigator and two supervisors monitored the process, while customized patient‐centered interview questionnaires and data extraction questionnaires were adopted and used to collect information from patients via phone or in‐person interviews. Pretesting was done on 10 patients prior to the study and appropriate modifications were made based on the results. Each eligible patient's chart was given a unique code number before data collection. Information such as socio‐demographic status, economic status, stage at presentation, and location of disease were collected. The patients were followed every 3 months for treatment details, PFS, and mortality rate.

### 2.10 Data analysis

After checking completeness, data entered into Epidata version 4.60 and then transferred to Statistical Package for Social Sciences (SPSS) for further analysis. Descriptive summaries, frequency tables, and graphs were used to describe study variables such as performance status, histologic type of cancer, and cancer stage at presentation. Chi‐square tests were used to identify the relationship between outcome variables, that is, survival and its predictors. To identify the survival of patients over time, we applied Kaplan Meier and tested its significance by using the log‐rank test. We applied Cox regression analysis to identify the predictor variables for the outcome variable. Those variables which had an association with a *p*‐value ≤.25 by univariate analysis were entered into multivariate Cox regression and *P* < .05 was considered statistically significant to establish the association between treatment outcome and predictor variables. And results were expressed using HR and 95% CI with the *p*‐value.

## 3 RESULTS

### 3.1 Sociodemographic characteristics

Among the 209 patients evaluated, the median age for diagnosis of CRC was 50 years (range, 16–90). A 25% of the patients were below the age of 38 years, and 75% of the patients were below the age of 60 years. Majority of the patients were Orthodox Christian by religion, accounting for 66% (*n* = 138), followed by Protestant (17.2%, *n* = 36) and Muslim (15.8%, *n* = 33). Most of the patients were from Addis Ababa, comprising 55% (*n* = 115), followed by Oromia and Amhara regions, which accounted for 23.4% (*n* = 49) and 11% (*n* = 23), respectively. A total of 39 (18.7%) patients had preexisting comorbidities, with hypertension being recorded in 20 (9.5%) patients, and diabetes being recorded in 14 (6.7%) patients. Alcohol usage was reported in 32 (15.3%) participants, and history of smoking was reported in 11 (5.2%) of the patients (Table 1).

**TABLE 1:** Sociodemographic characteristics of colorectal cancer patients seen at Tikur Anbessa Specialized Hospital, January 1, 2020 to September 10, 2022, Ethiopia.

Characteristics		Frequency	Percentage (%)
Age in years	<30	21	10.05
	30–39	41	19.6
	40–49	41	19.6
	50–59	41	19.6
	61–69	43	20.5
	>70	22	10.5
Gender	Male	119	56.9
	Female	90	43.1
Educational status	Can not read and write	45	21.5
	Read and write	32	15.3
	Primary education	43	20.6
	Secondary education	39	18.7
	College and above	50	23.9
Monthly income ETB	Below 1000	7	5.5
	1000–5000	97	75.8
	>5001–10 000	24	18,8
Comorbidity	Yes	39	18.7
	No	170	81.3
Family history	No	209	100
Marital status	Single	25	12
	Married	175	83.7
	Widowed/widower/divorced	9	4.3
Occupation	Farmer	27	12.9
	House wife	63	30.1
	Civil servant in gov't office	46	22
	NGO/private work	16	7.7
	Others^a^	57	27.2
Habits	Yes	46	22
	No	163	78

Abbreviations: ETB, Ethiopian Birr; NGO, Non‐Governmental Organization.

^a^ A day laborer, guard men, carpenter, students, and no job.

### 3.2 Clinical characteristics

Among the total 209 patients, 128 (61.2%) had rectal cancer, while 81 (38.8%) had colonic cancer. Nearly two‐thirds (134; 64.1%) of the patients had presented with bowel habit changes (constipation, diarrhea, or tenesmus) and a similar number presented with rectal bleeding. Of the 128 rectal cancer patients, 117 (91.4%) presented with rectal bleeding, and 82 (64%) had bowel habit changes. Only 20.1% of the 81 colonic cancer patients presented with rectal bleeding. Equal numbers (52 each) of colon and rectal cancer patients had presented with abdominal pain. At initial presentation, 49 (38.3%) rectal and 29 (35.8%) colonic cancer patients had weight loss. A total of 56 (26.8%) patients had presented with bowel obstruction symptoms at presentation; 34 of them were of colonic origin, while the rest were rectal. Masses on digital rectal examination were present in 82.5% of the rectal cancer patients. Two rectal cancer patients had inguinal lymphadenopathy, while 3 colonic cancer patients had supraclavicular lymphadenopathy (Tables 2 and 3).

**TABLE 2:** Clinical characteristics of colorectal cancer patients seen at Tikur Anbessa Specialized Hospital, January 1, 2020 to September 10, 2022, Ethiopia.

Clinical characteristics	Category	Frequency	Percentage (%)
Tumor location	Colon	81	38.8
	Rectum	128	61.2
Hemoglobin	Hgb ≤12.5	102	48.8
	Hgb >12.5	105	51.2
Common presenting symptoms	Bowel habit change	134	64.1
	Rectal bleeding	134	64.1
	Abdominal pain	114	54.5
	Weight loss	78	37.3
	Bowel obstruction	56	26.79
	Rectal discharge	41	19.6
	Others^a^	10	4.8
Performance status	ECOG‐0	2	1
	ECOG‐I	168	80.4
	ECOG‐II	31	14.8
	ECOG‐III	8	3.8
Histologic types	Adenocarcinoma	179	85.6
	Mucinous	14	6.7
	Signet ring	13	6.2
	Others^b^	3	1.5

Abbreviations: ECOG, Eastern Cooperative Oncology Group; Hgb, hemoglobulin.

^a^ Abdominal swelling, cough, and easy fatigability.

^b^ Neuroendocrine.

**TABLE 3:** Cox regression hazard model for risk factor associated with overall survival among colorectal cancer patient attending Tikur Anbessa Specialized Hospital from January 1, 2020 to September 10, 2022, Ethiopia.

Variables	Crosstab		Unadjusted				Adjusted			
	Censored	Death	HR	95% CI		*p*‐value	HR	95% CI		*P*‐value
*Age*										
≤40	21	46	1							
>40	47	95	. 93	.65	1.31	.671	1.53	1.02	2.29	.040*
*Educational level*										
College and higher	24	26	1				1			
Can not read and write, read, and write	20	51	1.95	1.22	3.10	.005	1.48	0.89	2.45	.134
Primary school	12	31	1.80	1.06	3.04	.027	2.01	1.15	3.53	.015*
Secondary school	12	27	1.78	1.04	3.06	.036	2.20	1.24	3.90	.007*
*ECOG*										
0–I	63	107	1				1			
II–III	5	34	2.18	1.47	3.21	.000	1.60	1.03	2.48	.035*
*Hgb at presentation*										
>12.5			1				1			
≤12.5	26	76	1.59	1.14	2.22	.006	1.55	1.06	2.25	.022*
*T‐staging*										
T‐1/2	11	6	1				1			
T‐3	37	42	1.71	.73	4.03	.217	2.42	.92	6.34	.072
T4	20	93	4.43	1.93	10.15	.000	6.05	2.28	16.02	.000**
*N‐staging*										
N‐0	23	18	1				1			
N‐1	15	43	2.12	1.22	3.67	.008	.99	.52	1.93	.998
N‐2	19	75	2.83	1.67	4.75	.000	1.07	.57	2.02	.832
Nx	11	5	.64	.24	1.73	.378	1.58	.55	4.56	.393
*Group staging*						.000				
Stages I–II	33	11	1				1			
Stage‐III	23	49	4.12	2.13	7.95	.000	3.65	1.54	8.62	.003*
Stage‐IV	12	81	8.25	4.36	15.57	.000	8.53	3.77	19.25	.000**
*Chemotherapy*										
Planed and given	55	80	1				1			
Planned but not given	9	54	2.35	1.66	3.34	.000	2.11	1.44	3.09	.000**
Not planned not given	4	7	1.39	.64	3.02	.401	2.84	1.17	6.90	.021*

Abbreviations: CI, confidence interval; ECOG, Eastern Cooperative Oncology Group; Hgb, hemoglobulin; HR, hazard ratio; N, lymph node staging; *P*, significant value; T, tumor staging.

* Statistically significant in the multivariate cox‐regression with a *p*‐value of <.05.

** Highly statistically significant in the multivariate cox‐regression with a *P*‐value of <.001.

### 3.3 Stage at presentation

**FIGURE 1:**
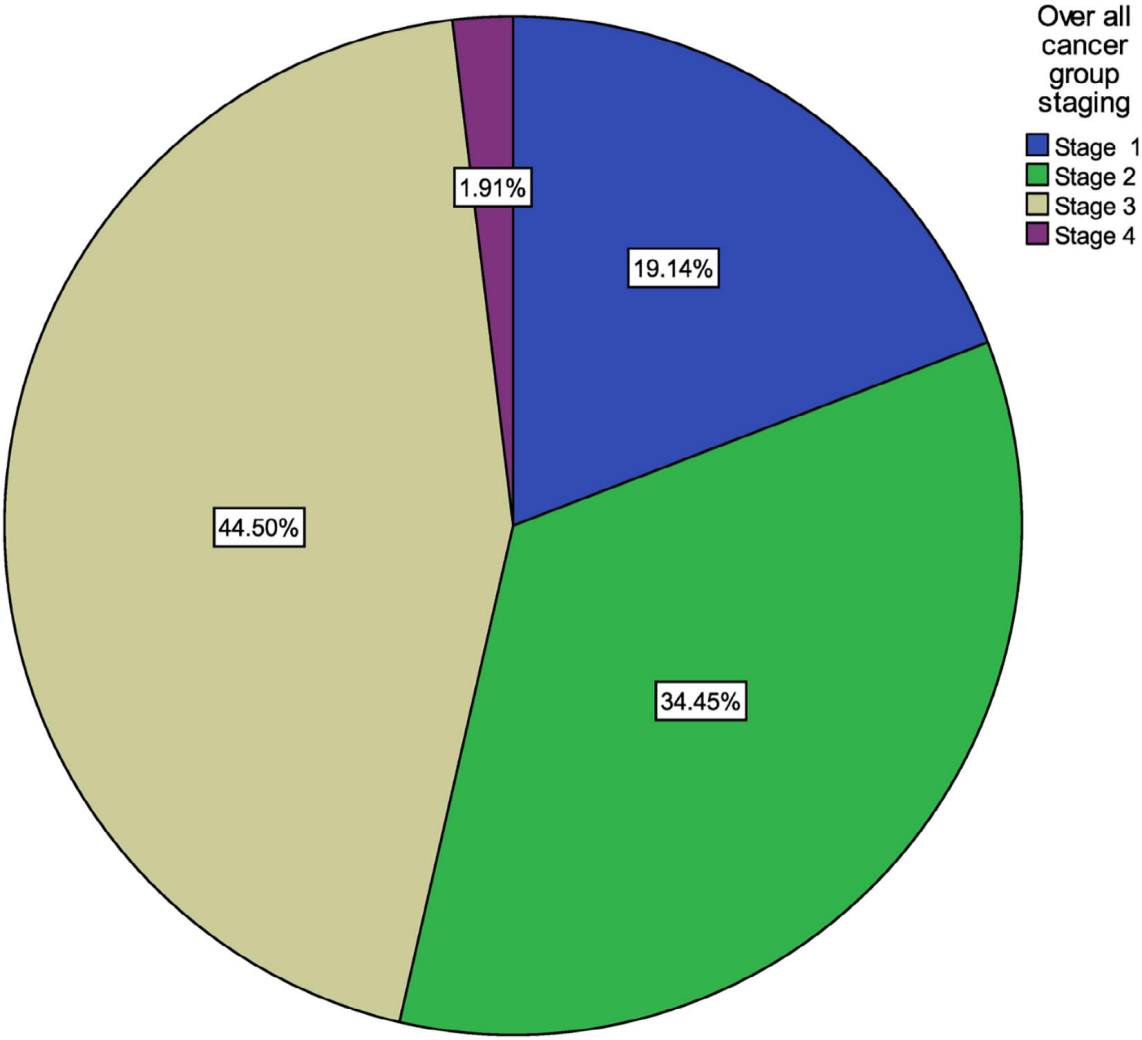
Stage of cancer at presentation for colorectal cancer patients in Tikur Anbessa Specialized Hospital, January 1, 2020 to September 10, 2022, Ethiopia.

More than three‐fourths (79.4%) of the patients were found to have advanced disease (stages III/IV). Of the 104 patients who underwent surgery, 56 (53.8%) were not staged preoperatively (Figure 1).

### 3.4 Treatment delivered

The treatment status of 209 patients was evaluated, of which 138 (66%) had an indication for curative surgery. Of these, 34 (16.3%) did not receive the planned treatment. Most of the procedures were performed on an elective basis 69(66.3%), with 35 (33.7%) undergoing emergency surgery and 26 (12.4%) having a palliative diversion stoma. Of those who needed CT, 63 (30.1%) did not receive the planned treatment due to a long waiting list for treatment and financial constraints and 53 (39.3%) did not complete their recommended regimens related to a number of factors, including deteriorating performance and tumor progression. Of the 77 rectal cancer patients booked for RT with curative intent, only 15 (7.2%) received the treatment. In total, 56 patients (26.8%) did not receive any oncologic treatment (Table 4).

**TABLE 4:** Treatment profile of colorectal cancer patients seen at Tikur Anbessa Specialized Hospital, January 1, 2020 to September 10, 2022, Ethiopia.

Type of treatment given	Category	Frequency	Percentage (%)
Surgery (*n* = 209)	Planned and done	104	49.8
	Planned but not done	34	16.3
	Not planned and not done	45	21.5
	Palliative diversion stoma	26	12.4
Timing of surgery (*n* = 104)	Elective	69	66.3
	Emergency	35	33.7
Chemotherapy (*n* = 209)	Planned and given	135	64.6
	Planned but not given	63	30.1
	Not planned and not given	11	5.3
Completed the prescribed cycles of CT (*n* = 135)	Yes	82	60.7
	No	53	39.3
RT/CCRT (*n* = 209)	Planned and given	15	7.2
	Planned but not given	62	29.7
	Not planned and not given	132	63.2
No treatment^a^ (*n* = 209)	No	56	26.8

Abbreviations: CT, chemotherapy; RT/CCRT, radiotherapy or concurrent chemoradiotherapy.

^a^ No oncologic intervention at all in the course of patient management (surgery, CT, RT, CCRT).

### 3.5 Survival

**FIGURE 2:**
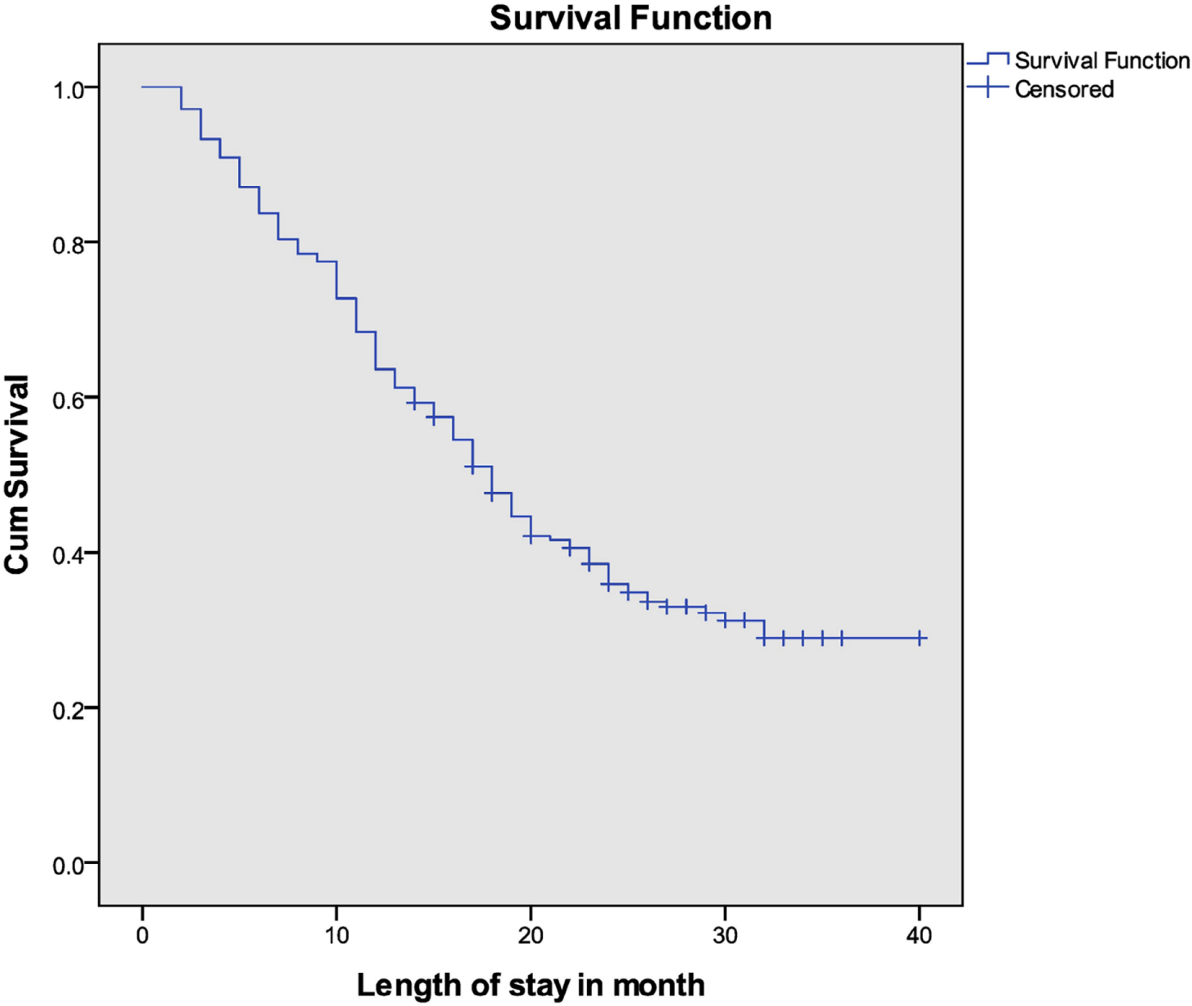
Kaplan Meir overall survival function of colorectal cancer patients in Tikur Anbessa Specialized Hospital, January 1, 2020 to September 10, 2022, Ethiopia.

**FIGURE 3:**
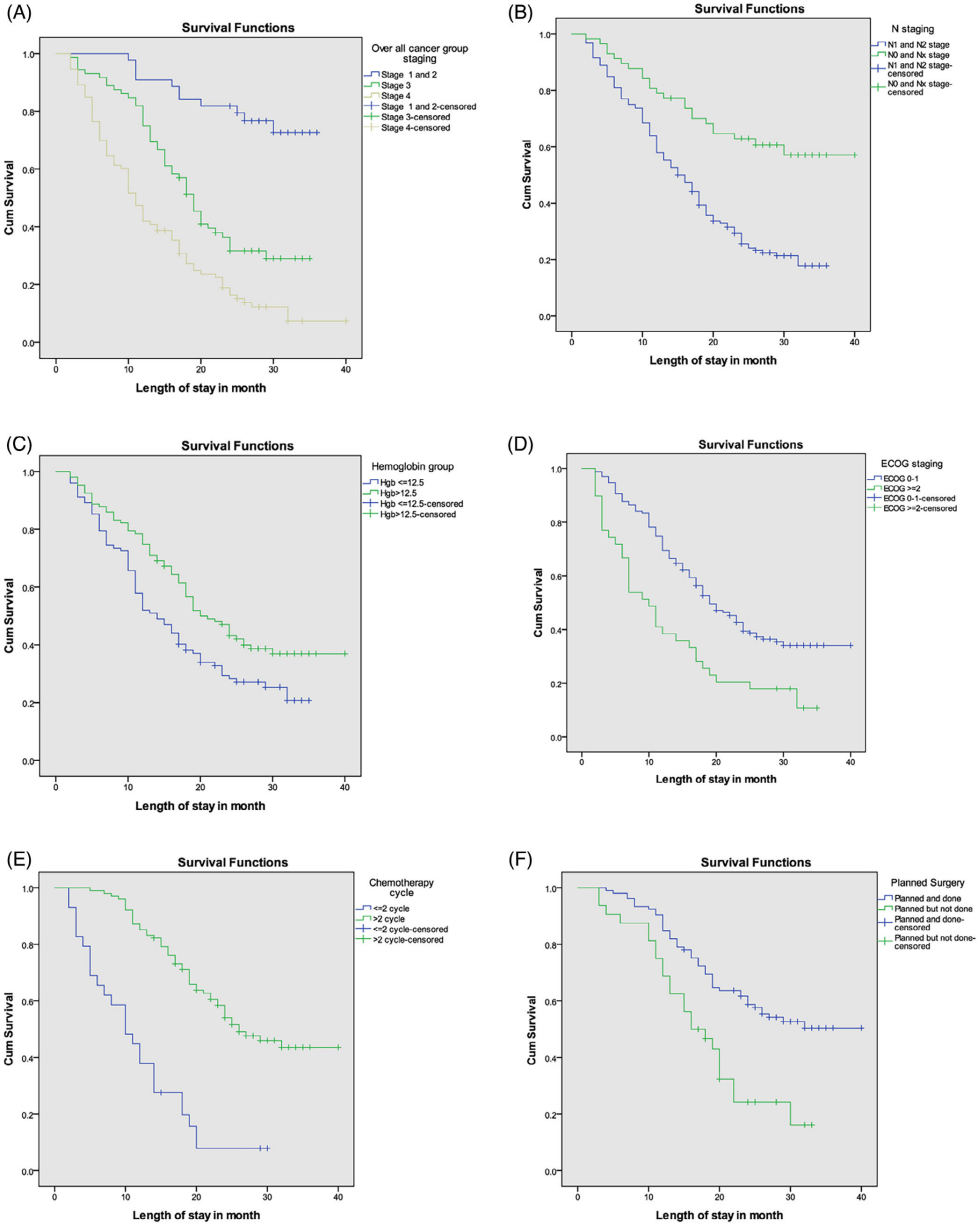
(A–F) Factors associated with survival of colorectal cancer Patients with log‐rank test Seen at Tikur Anbessa Specialized hospital, January 1, 2020 to September 10, 2022, Ethiopia.

The median follow‐up duration was 20 months, with a minimum of 14 months and a maximum of 40 months. Overall, 141 patients (67.46%) died with a 95% CI (61%, 74%) between the time of diagnosis and the time of data analysis. Of these, 11 (25%) of the 44 patients with early‐stage disease, 49 (68.05%) of 72 stage‐III patients, and 81 (87.09%) of 93 stage‐IV patients died. The median OS was 17 months (SD, ±10), ranging from 2 to 32 months (Figure 2.). The 1‐year OS for our patients was 63.16%, with a 95% CI (56.23%, 69.29%). In terms of staging, the 1‐year survival rates were 91%, 69%, and 41% for stages I/II, III, and IV, respectively (Figure 3).

The median survival (MS) for patients with good performance status at presentation was 19 months, while for those with poor performance status, it was 10 months. Patients with early stage disease had a MS of 33.2 months, whereas those with advanced stage disease had a MS of 19 months. The MS for those who underwent the planned surgical intervention was 26 months, compared to 16 months for those who did not. In the CT group, the MS was 22 months, while those who did not receive the recommended CT regimen had a MS of 12 months (Table 5).

**TABLE 5:** Factors associated with survival of colorectal cancer patients seen at Tikur Anbessa Specialized Hospital using log rank test, January 1, 2020 to September 10, 2022, Ethiopia

Variable	Category	Cross tab		Median OS (95% CI)	*P*‐value
		Censored	Dead		
Gender	Male	42	77	19.0 (14.9, 23.0)	.393
	Female	26	64	17.0 (14.7, 19.3)	
Age	≤40 years	21	46	17.0 (15.3, 18.7)	.697
	>40 years	47	95	19.0 (15.6, 22.4)	
Marital status	Not married	12	22	17.0 (13.2, 20.9)	.887
	Married	56	119	18.0 (15.4, 20.5)	
Education level	Not read at all, read and write	20	57	16.0 (19.8, 12.2)	.033*
	Primary school	12	31	17.0 (11.5, 22.5)	
	Secondary school	12	27	17.0 (11.2, 22.8)	
	College and above	24	26	30.0 (21.3, 38.7)	
Region	Addis Ababa	40	75	19.0 (16.2, 21.8)	.296
	Out of Addis Ababa	28	66	17.0 (14.5, 19.5)	
Comorbidity	Yes	14	25	20.0 (11.8, 28.2)	.483
	No	54	116	17.0 (19.2, 14.8)	
ECOG	ECOG 0–1	63	107	19.0 (15.9, 22.3)	.001**
	ECOG ≥ 2	5	34	10.0 (6.9, 13.1)	
Hemoglobin level	Hgb ≤ 12.5	26	76	14.0 (10.5, 17.5)	.006*
	Hgb > 12.5	42	65	20.0 (15.8, 24.2)	
Site of cancer	Colon	30	50	18.0 (13.7, 22.3)	.331
	Rectum	38	91	18.0 (15.5, 20.5)	
Group staging	Stage‐I/II	33	11	33.2 (30.7, 35.8)	.001**
	Stage‐III	23	49	19.0 (16.8, 21.2)	
	Stage‐IV	12	81	11.0 (9.3, 12.7)	
T staging	T‐2	11	6	26.3 (23.6, 32.1)	.001**
	T‐3	37	42	25.0 (17.7. 32.3)	
	T‐4	20	93	12.0 (9.2, 14.8)	
N staging	N1 and N2	34	118	15.0 (12.2, 17.8)	.001**
	N0 and Nx	34	23	25.0 (22.3, 29.6)	
Surgery	Planned and done	56	49	26.0 (24.2, 33.1)	.001**
	Planned but not done	8	24	16.0 (10.7, 21.3)	
Surgery timing	Elective	41	29	32.0 (27.7, 33.2)	.043*
	Emergency	15	20	24.0 (15.8, 32.2)	
Chemotherapy	Planned and given	55	80	22.0 (17.9, 26.1)	.001**
	Planned but not given	9	54	12.0 (9.7, 14.3)	
Completed cycles of the prescribed CT	Yes	45	37	32.0 (30.4, 35.4)	.001**
	No	10	43	12.0 (9.3, 14.7)	
The overall duration of CT	≤6 months	38	20	31.0 (28.9, 34.8)	.012*
	>6 months	12	20	24.0 (21.5, 26.5)	
Radiation or chemoradiation	Planned and given	8	7	32.0 (10.4, 53.6)	.114
	Planned but not given	21	41	19.0 (16.8, 21.2)	
Number of CT cycle	≤2 cycle	3	26	10.0 (6.0, 13.9)	.001**
	>2 cycle	49	52	26.0 (19.9, 32.1)	

Abbreviations: CI, confidence interval; CT, chemotherapy; ECOG, Eastern Cooperative Oncology Group; Hgb, hemoglobulin; OS, overall survival.

* Statistically significant in the log‐rank test with a *p*‐value of <.05.

** Highly statistically significant in log‐rank test with a *P*‐value of <.001.

**FIGURE 4:**
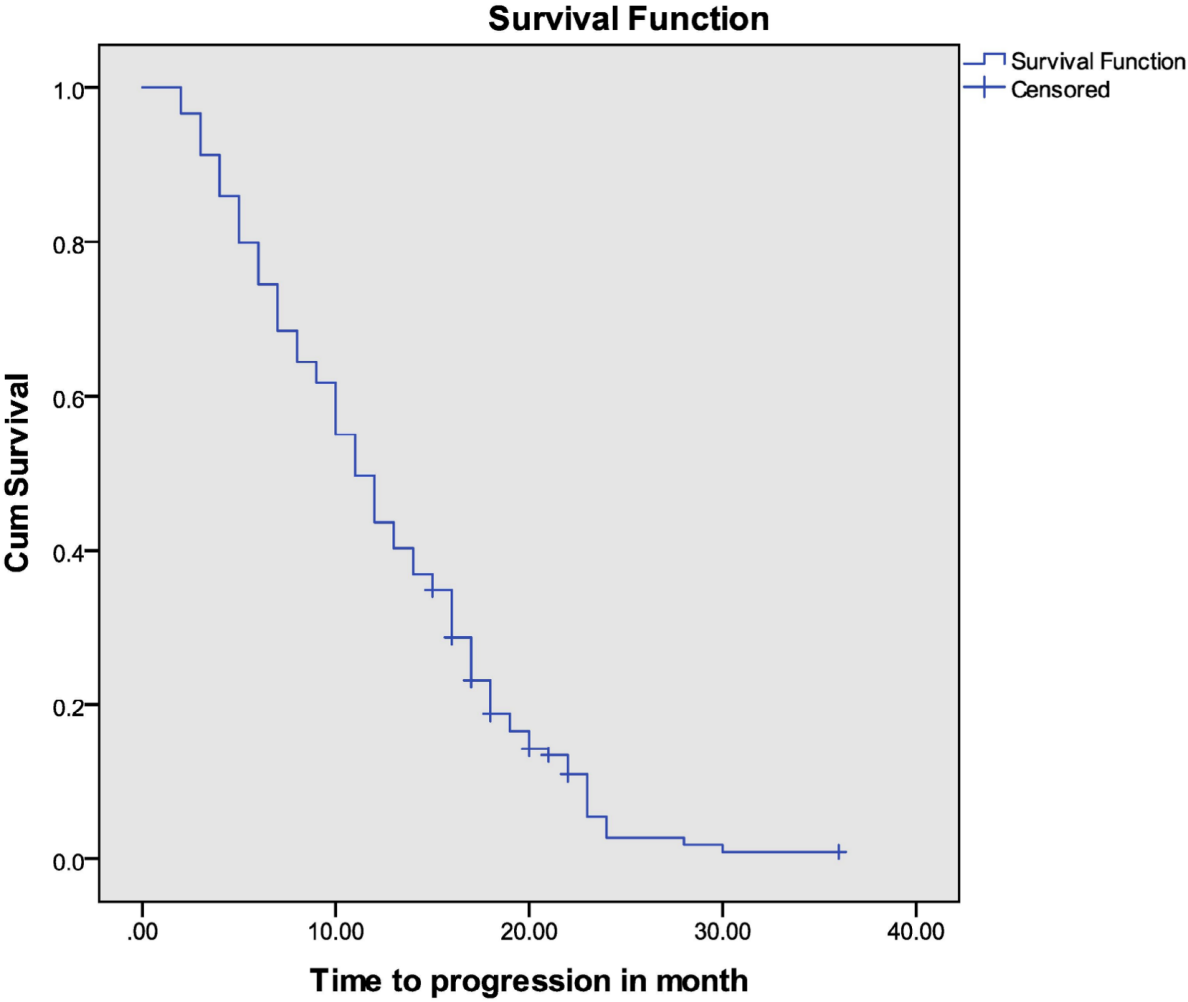
Kaplan Meir progression‐free survival function of colorectal patients in Tikur Anbessa Specialized Hospital, January 1, 2020 to September 10, 2022, Ethiopia.

An analysis of PFS was conducted on 205 patients who met the criteria. Of these, 147 (71.70%) patients experienced progression at some point during the study period. Among these, death was considered progression for 96 (45.93%) patients, and 109 (52.15%) patients experienced either local or distant recurrence. Multiple site (local, liver, lung, bone, etc.) recurrence was observed in 19 patients, followed by liver‐only metastases in 15 patients, and local recurrence in 10 patients. The PFS of these 205 participants ranged from 2 to 20 months, with a median of 11 months (SD ± 0.7) (See Figure 4).

### 3.6 Factors affecting overall survival

Age over 40 years increases the risk of death by 53% when compared to younger age groups (HR = 1.53, 95% CI, 1.02–2.29, *P* < .040). Poor performance status (ECOG‐II & III) was associated with a 60% greater likelihood of dying than good functional status (HR = 1.60, 95% CI, 1.03–2.48, *P* < .035). Hemoglobin (Hgb) levels less than 12.5 g/dL were associated with a 1.5‐fold higher risk of death (HR = 1.55, 1.03–2.08, *P* < .035) than those without anemia. Tumor depth of invasion was also an independent predictor of survival, with T‐4 disease associated with a 6‐fold increased risk of mortality compared to T1 and T2 disease (HR = 6.05, 95% CI, 2.28–16.02, *P* < .001). Metastases at diagnosis was associated with an eight‐fold increased risk of death compared to early‐stage cancer (stage I/II, HR = 8.53, 95% CI, 3.77–19.25, *P* < .001). Inability to deliver recommended surgery and CT were also poor prognostic factors (See Table 3, Figure 3A–F).

## 4 DISCUSSION

The findings of this study are of great importance and novelty as they provide insight into the treatment type and outcomes of CRC patients in Ethiopia. The results highlight the need for improved access to cancer treatment in the region, given the poor survival rate due to advanced stages of the disease and a lack of timely treatment. Additionally, the study identified prognostic factors for survival, which are important for developing strategies to improve CRC outcomes in Ethiopia.

In this study, 67.46% of patients died in a median follow‐up duration of 20 months, with a 1‐year OS rate of 63.13% (95% CI, 56.23%–69.29%). This is similar to a retrospective study in Ghana, where the 1‐year survival rate was 64%.^29^ Comparatively to other regions, SSA has a low rate of CRC survival, where a meta‐analysis found the 1‐year survival is 74%, and even lower in lower income countries.^32^ Our finding is far lower than many other studies performed in different parts of the world, such as a study from Brazil, where the 5‐year OS was 63.5% and 60.6% for colonic and rectal malignancies, respectively,^33^ and a Chinese study where the 3‐ and 5‐year OS was 74% and 68%, respectively.^26^ In a study in Vietnam, the 1, 3, and 5 OS survival showed 84.7%, 56%, and 45%, respectively,^34^ which are greater than 1‐year survival of our patients and this demonstrates the need of addressing the situation, which necessitates the involvement of all stakeholders.

The MS time observed in our patient is 17 months, and 11 months for stage IV disease, which is comparable to a previous study in Ghana, MS which is 15 months, where lack of treatment delivery was the main reason for poor survival.^29^ However, for stage ‐III patients MS we found was 19 months, which is lower than a report from Nigeria, MS for stage‐III is 24 months, due to curative treatment delivery being relatively better in such locally advanced cases.^31^ In comparison to the survival rate of Vietnamese patients, the MS time was 48.59 months (39.34–57.93 months).^34^ This stark disparity resulted from varying stage distribution and type of treatment delivery.

Progression free survival is established as a surrogate marker for OS.^35^ In present study, the median PFS was 11 months, and only 58 (27.8%) patients have no progression at the end of the study period. For stage II and stage III cases, median PFS was 16 and 13 months respectively. However, the 5 years PFS of stage II/III patients in a study done in Oregon is 90%, with proper adjuvant CT being identified as a protective factor for recurrence.^36^ It is true for our patients who have not received the appropriate treatment recommendations, which results in early disease recurrence or death. The 5 years PFS of stage‐II disease in Italy is 78.4%, which showed the relevance of timely initiation of adjuvant CT.^37^

There are several factors that could affect patient survival seen in many studies. In our assessment, a significant number of patients, 67 (32.06%), were under the age of 40 at the time of diagnosis. We found that the probability of death increased with age greater than 40 years (HR = 1.53, 95% CI, 1.02–2.29, *p* < .040). This is consistent with a study from Canada, which found that age younger than 45 years was an independent predictor of better survival.^38^ However, early‐onset (age < 50) CRC has a poor prognosis due to its advanced stage at diagnosis and poorly differentiated histology.^39^ Colonoscopy screening at age 45 and flexible sigmoidoscopy screening at age 40 is beneficial in identifying more patients with early‐onset CRC in individuals who are at average risk.^40^ The difference in prognosis between the two groups could be attributed to the aggressive nature of tumor biology in the younger age group, whereas multiple comorbidities and frailty in the older age group resulted in a barrier to initiating recommended cancer therapy.

Despite advances in cancer treatment, socioeconomic inequities continue to play a significant impact on all types of cancer survival. Socioeconomically disadvantaged groups are at a higher risk of death.^41^ In our investigation, the MS time for patients with a college or higher education was 30 months (95% CI, 21.3–38.7, log‐rank: *p* < .033) and 17 months for those with primary education. In multivariate analysis, high school graduates have a doubled risk of death (HR = 2.2, 95% CI, 1.24–3.90, *p* < .007) compared to college and above graduates. This is consistent with a Swedish study, patients with lesser levels of education had worse 5‐year relative survival (57.9% vs. 63.8% in colon cancer, 58.7%–69.1% in rectal cancer).^42^ Better health awareness and early diagnosis, higher income, and good follow‐up care could all explain this.

Advanced stage is the most important predictor of CRC patient outcome.^43^ In our study, the stage of the disease is an independent prognostic factor for lower survival (HR = 8.53, 95% CI, 3.77, 19.25, *p* < .000) for the stage‐IV disease relative to early‐stage disease (stage 1/II). This is confirmed in earlier studies done on Chinese^26^ and Iranian^44^ patients. A comprehensive population‐based study conducted in four European nations (Denmark, England, Norway, and Sweden) also found that the stage of the disease is the most important predictor of CRC survival.^27^ Furthermore, various factors influence the stage of the disease including sociodemographic characteristics of patient, biology of the disease and delay in diagnosis.

Poor performance status is an indication of cancer burden and has been shown to reduce survival for a wide range of reasons.^43^ In our study, patients with an ECOG of II/III had a risk of death increased by almost 60% (HR = 1.60, 95% CI, 1.03, 2.48, *p* < .035). These patients are unable to tolerate CT and/or RT treatment and they end up receiving supportive palliative care. Hence, leading to higher mortality in this group.

Low Hgb level in cancer patients can be caused by cancer itself or by a combination of factors; however, it has a significant impact on cancer treatment and patient survival in the long run due to its ability to predict advanced disease.^45^ In this study, we observed that Hgb levels less than or equal to 12.5 mg/dL were related to poor OS, (HR = 1.55, 95% CI: 1.06–2.25, *p* < .022). This is consistent with a study from Finland, where preoperative normocytic anemia is associated with a generally lower survival rate (HR = 1.61, 95% CI, 1.07–2.42, *p* < .023).^46^ Furthermore, the impact of lower Hgb level is more pronounced in black African Americans.^47^

After cancer is diagnosed, treatment should be initiated as soon as possible considering tumor progression with time as well as psychological stress in patients. Unfortunately, in our investigation, 30.1% patients did not receive the prescribed CT at all, and only 15 patients had received RT as recommended among the 77 rectal cancer patients due to prolonged waiting time for RT as TASH has been the only RT providing cancer center in the country until recently. In multivariate cox regression, lack of CT was statistically associated with poor survival by two times (HR = 2.11, 95% CI, 1.44–3.09, *p* < .000). This is consistent with a study from Ghana, where the protective effect of CT was observed (HR = 0.23, 95% CI, 0.13–0.41, *p* < .0001).^29^ In a Nigerian study, patients who got the recommended treatment had a significantly higher MS (25 vs. 7 months).^31^ Due to a lack of resources in Ethiopia, 56 (26.8%) CRC patients did not receive any form of oncologic intervention. This is the major gap observed in the management of CRC patients and it can be the primary explanation for the high case fatality rate in our setup. In contrast, most patients with locally advanced resectable or metastatic cancers in developed countries undergo standard‐of‐care treatments such as surgery, CT, and RT as deemed appropriate given well‐equipped cancer treatment centers and experienced healthcare professionals, resulting in a higher cure rate.^17^, ^28^ In Norway, the median waiting time for RT is 14–50 days,^48^ while in Taiwan, about 90.5% of CRC patients received the recommended oncologic treatment within 30 days.^47^ Our practice is not consistent with any of the existing studies. This highlights the need for improved access to cancer treatment in Ethiopia.

## 5 CONCLUSION

Our patient's 1‐year survival rate is low, and the overall mortality rate is high. Majority of the patients presented at an advanced stage and significant number of patients were unable to receive the recommended treatment due to lack of resources. Older age, low Hgb level, lower education level, advanced stage upon presentation, poor functional status, and an inability to receive recommended treatments all contributed to the poor survival rate. This study demonstrates that CRC treatment and outcomes in tertiary cancer center in Ethiopia differ significantly from other comparative studies. To improve the treatment outcomes of CRC patient, we recommend that initiatives should be taken to increase awareness of health‐seeking behavior within the community for early diagnosis and to ensure that all treatment modalities are available to patients by prioritizing cancer treatment center expansion and capacity building.

### 5.1 Strength of the study

The strength of this study lies in its prospective nature and the fact that it was conducted at the largest and only comprehensive oncology center in Ethiopia.

### 5.2 Limitations of the study

The COVID‐19 pandemic presented a limitation to the study as patient follow‐up became challenging. This resulted in many patients being excluded from the final analysis, thus limiting the exploration of further prognostic factors in the regression analysis due to the small proportions of patients in the cohorts.

## AUTHOR CONTRIBUTIONS

**Girum Tessema Zingeta:** Conceptualization (lead); data curation (equal); formal analysis (equal); funding acquisition (equal); investigation (lead); methodology (lead); project administration (lead); resources (lead); supervision (equal); validation (equal); writing – original draft (lead); writing – review and editing (lead). **Yohannes T. Worku:** Conceptualization (supporting); data curation (equal); investigation (supporting); writing – review and editing (supporting). **Assefa Getachew:** Conceptualization (equal); funding acquisition (equal); methodology (supporting); supervision (equal); writing – review and editing (equal). **Jilcha Diribi Feyisa:** Methodology (equal); writing – original draft (supporting); writing – review and editing (equal). **Hawi Furgassa:** Data curation (supporting); methodology (supporting); writing – review and editing (equal). **Winini Belay:** Data curation (supporting); formal analysis (supporting); writing – review and editing (supporting). **Tariku Mengesha:** Formal analysis (equal); methodology (supporting); writing – review and editing (supporting). **Ahmedin Jemal:** Conceptualization (equal); funding acquisition (equal); methodology (equal); project administration (equal); supervision (equal); validation (equal); writing – review and editing (equal). **Mathewos Assefa:** Conceptualization (equal); funding acquisition (equal); investigation (equal); methodology (equal); project administration (equal); resources (equal); supervision (equal); writing – original draft (supporting); writing – review and editing (equal).

## FUNDING INFORMATION

The Addis Ababa University School of Public Health received funding for this work through an American Cancer Society‐supported colorectal cancer thematic research project award. However, they are not involved in the methodology, data collecting, analysis, or manuscript writing.

## CONFLICT OF INTEREST STATEMENT

The authors have stated explicitly that there are no conflicts of interest in connection with this article.

## ETHICS STATEMENT

Ethical clearance was obtained from Addis Ababa University Department of Clinical Oncology. Written informed consent to participate in the study was obtained from all patients. Confidentiality of the information was maintained throughout the study by excluding names as identification in the data extraction form and the data used only for the purpose of the conducted study.

## Data Availability

The data used for this research will be available upon request.
